# A Novel Spectral Barcoding and Classification Approach for Complex Biological Samples Using Multiexcitation Raman Spectroscopy (MX-Raman)

**DOI:** 10.1021/acs.analchem.5c00776

**Published:** 2025-06-03

**Authors:** George Devitt, Niall Hanrahan, Miguel Ramírez Moreno, Amrit Mudher, Sumeet Mahajan

**Affiliations:** † School of Biological Sciences, 7423 University of Southampton, Highfield Campus, SO17 1BJ Southampton, U.K.; ‡ School of Chemistry and Chemical Engineering, University of Southampton, Highfield Campus, SO17 1BJ Southampton, U.K.; § Institute for Life Sciences, University of Southampton, Highfield Campus, SO17 1BJ Southampton, U.K.

## Abstract

We report the development and application of a novel spectral barcoding approach that exploits our multiexcitation (MX) Raman spectroscopy-based methodology for improved label-free detection and classification of complex biological samples. To develop our improved MX-Raman methodology, we utilized post-mortem brain tissue from several neurodegenerative diseases (NDDs) that have considerable clinical overlap. For improving our methodology we used three sources of spectral information arising from distinct physical phenomena to assess which was most important for NDD classification. Spectral measurements utilized combinations of data from multiple, distinct excitation laser wavelengths and polarization states to differentially probe molecular vibrations and autofluorescence signals. We demonstrate that the more informative MX-Raman (532 nm–785 nm) spectra are classified with 96.7% accuracy on average, compared to conventional single-excitation Raman spectroscopy that resulted in 78.5% accuracy (532 nm) or 85.6% accuracy (785 nm) using linear discriminant analysis (LDA) on 5 NDD classes. By combining information from distinct laser polarizations we observed a nonsignificant increase in classification accuracy without the need of a second laser (785 nm–785 nm polarized), whereas combining Raman spectra with autofluorescence signals did not increase classification accuracy. Finally, by filtering out spectral features that were redundant for classification or not descriptive of disease class, we engineered spectral barcodes consisting of a minimal subset of highly disease-specific MX-Raman features that improved the unsupervised and cross-validated clustering of MX-Raman spectra. The results demonstrate that increasing spectral information content using our optical MX-Raman methodology enables enhanced identification and distinction of complex biological samples but only when that information is independent and descriptive of class. The future translation of such technology to biofluids could support diagnosis and stratification of patients living with dementia and potentially other clinical conditions such as cancer and infectious disease.

## Graphical abstract

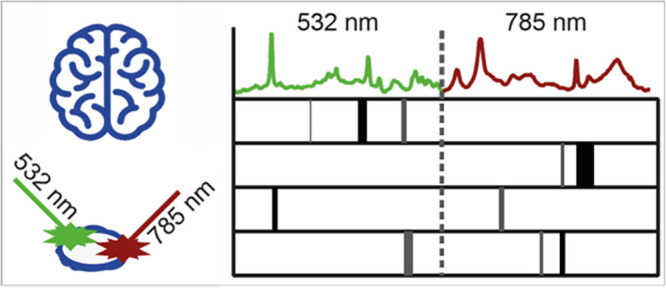

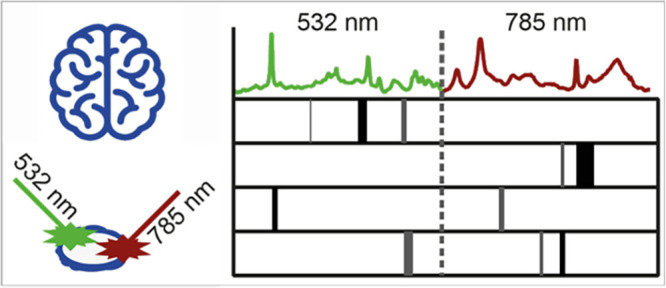

## Introduction

The amount of information required to accurately classify a sample scales with complexity for a given data set size. Maximizing information content and using different and independent features is essential to maximize the performance of classification models for multicomponent samples. Biological samples are complex by nature and contain multiple biomolecules including many different proteins, metabolites, lipids, nucleotides and sugars. This presents a huge challenge for a complete analysis to distinguish between diseases that are closely related or the subtypes of a given condition, as the compositional changes are likely to be minimal. For clinically overlapping diseases, single biomarkers are often not enough to achieve adequate and sensitive diagnoses.^1^ Omics-based technologies are being used to identify biomarkers, but they are often targeted and rely on known information obtained using sophisticated instrumentation. Using meta-omics (meta-genomics, proteomics, metabolomics) allows the unbiased detection of subtle changes, but at the expense of simplicity, speed and cost.^2^

A potential simple and elegant solution is Raman spectroscopy, which is an optical chemical characterization technique that can be carried out without any sample preparation and can provide readouts rapidly in a highly scalable and affordable manner.^3–5^ Raman spectroscopy probes the vibrational modes of molecules within a sample to provide a unique and label-free spectrum or “chemical fingerprint”.^6^ Raman active vibrational modes manifest as peaks in a spectrum wherein different peaks correspond to bonds and structural moieties within a molecule, as well as from different molecules within the sample.^7^ The rich information contained in a Raman spectrum represents the collective characteristics of a sample, making the technique holistic as opposed to reporting only specific analytes. Being label-free, it is also unbiased since all Raman active vibrations in molecules will be detected based on their strength of interaction, that is, their Raman cross-section.^8^ The measurement itself and the analysis can be performed within seconds or minutes, and the technique is amenable to automation and portability making it very attractive for in-field clinical applications.^9^

However, the holistic nature of Raman spectra also means that the inherent variation between individual complex biological samples may be subtle given that the constituent biomolecules such as proteins, lipids, DNA, sugars and metabolites are essentially made of similar bonds with overlapping vibrational frequencies. Hence, chemometric (multivariate) methods are often necessary to extract spectral differences between complex biosamples and often their stratification through unsupervised statistical methods, such as simple clustering, is difficult.

We investigate two ways to increase the differential analysis capability of the technique especially for complex and closely related samples. We first increase the information content by measuring the Raman spectra of samples by two different lasers. While Raman peak shifts are independent of laser excitation, the Raman cross-section is wavelength dependent, which is evident through the observation of preresonance and resonance Raman spectra.^8^ The wavelength dependence of the Raman cross-section is in addition to the λ^–4^ dependence characteristic of scattering processes.^10^ Moreover, Raman signals are polarized based on the symmetry of the vibrations.^11^ Thus, by using well separated distinct laser excitations and by using polarized detection we can get a much more characteristic fingerprint and, critically, more information than in a conventional Raman spectrum. We call this novel method multiexcitation Raman spectroscopy (MX-Raman). We have previously validated the utilization of distinct laser wavelengths enabling the differential enhancement of resonant molecular components to facilitate enhanced supervised classification of bacteria by the combination of chemical information.^8^ Apart from demonstrating the MX-Raman barcoding and intelligent feature engineering approach, here we develop the multiexcitation concept further additionally using polarized excitation for Raman and autofluorescence signals.

In addition to obtaining independent multivariate information such as with the MX-Raman technique, accurate classification of closely related samples requires appropriate data sets as well as computational methods that can deal with the subtle differences and correlated features that are prominent in Raman spectra of biological samples.^12^ Thus, traditional multivariate methods such as principal component analysis and linear discriminant analysis (PCA-LDA) have been used for classification of biological^13^ and clinical^14^ samples. Increasingly, machine learning is being applied to Raman spectroscopy data for classification of disease from clinical samples including cancer,^15^ bacterial infection^16^ and neurodegenerative disease.^17^ While reported classification accuracies are typically good (∼90%), they often rely on small sample numbers may decrease in larger, less defined cohorts, such as real-world populations where similar diseases and subtypes with overlapping clinical features are prevalent.^18^ It is therefore important to maximize discriminatory information in the Raman spectrum while minimizing noise and redundant information, which can decrease machine-learning (ML) classification performance and generalizability.^19^ Often, “black box” ML algorithms are applied to the whole Raman spectrum without any understanding of which spectral regions are responsible for classification, resulting in issues including overfitting and poor spectral assignment, raising doubts about the clinical translation of such models.^20^ We use intelligent feature engineering to overcomes the above issues including the finite number of clinical samples that are available for developing diagnostic methods.

In this work, we develop a novel MX-Raman spectral barcoding approach and provide the first validation of MX-Raman barcodes for the classification of complex biological samples (Figure 1). Specifically, we analyze the insoluble tissue fraction isolated from the post-mortem brains of patients with several neurodegenerative diseases (NDDs) that have considerable clinical overlap (Alzheimer’s disease, AD; Pick’s disease, PiD; progressive supranuclear palsy, PSP; and corticobasal degeneration, CBD) and non-neurodegenerative controls (*n* = 3 per each group). We show that MX-Raman spectra from each of these samples can be classified with less error than conventional (single-excitation) Raman spectroscopy, while identifying the spectral frequencies that are responsible for classification enabling the assignment of spectral biomarkers or “barcodes”. Our proof-of-concept study highlights the potential utility of Raman spectroscopy-based methods such as MX-Raman barcoding to the diagnosis and stratification of NDDs.

**Figure 1:**
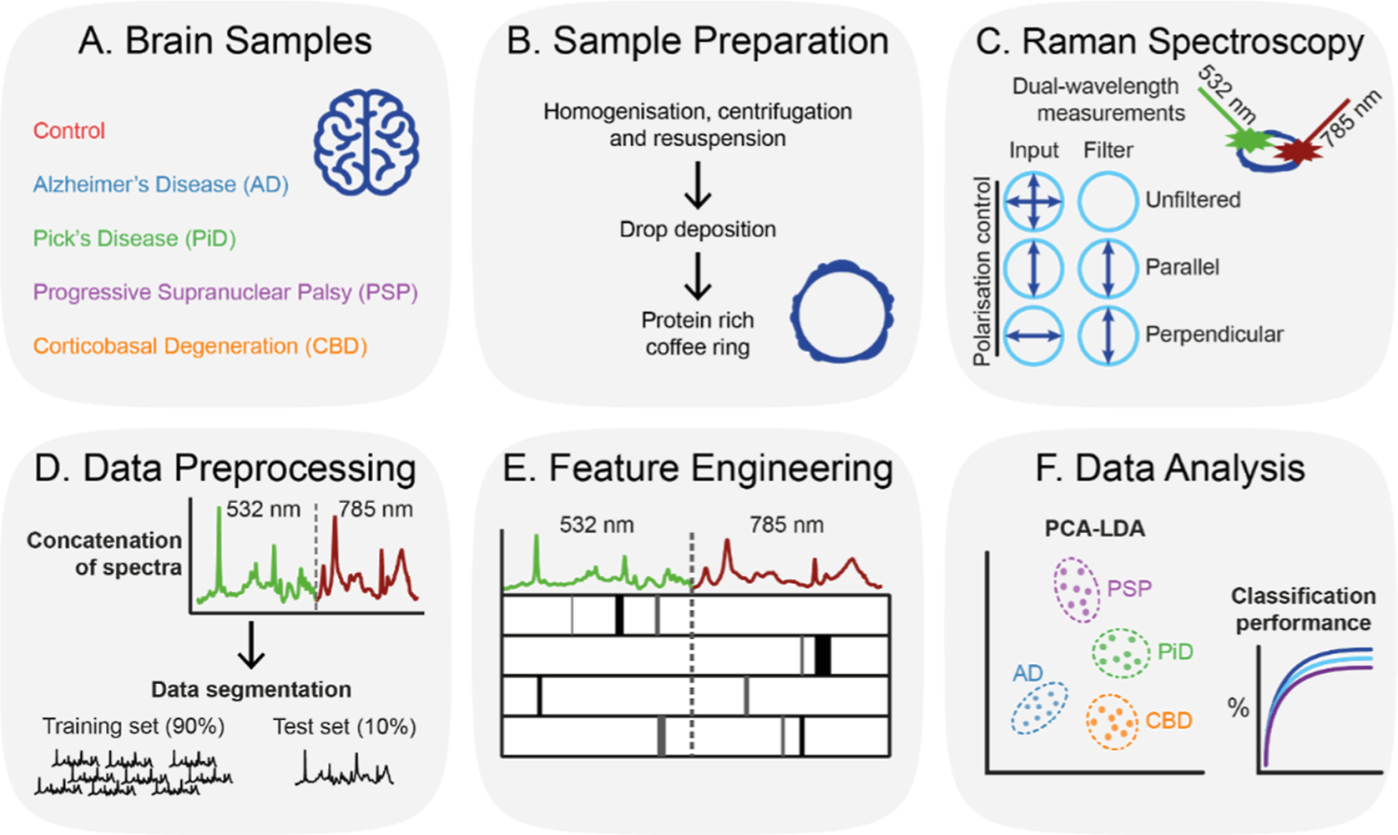
Schematic of workflow. (A) Post-mortem brain samples used in this study. (B) Sample preparation for Raman spectroscopy. (C) Raman spectroscopy configurations. (D) Data Preprocessing for MX-Raman and classification. (E) Feature engineering for spectral barcode development. (F) Data analysis including clustering and classification.

## Results

### Increasing Information Content Using MX-Raman

#### Increasing Information Content Using Multiple Lasers

Classification accuracy was assessed for preprocessed Raman fingerprints from 532 and 785 nm excitations individually and in combination for MX-Raman. The average 532 nm fingerprint for each class is shown in Figure 2A. The 532 nm fingerprints are notably different from the 785 nm fingerprints shown in Figure 2B. While the Raman cross-section of vibrational modes has a wavelength dependence, the observed difference is caused by the resonant and preresonant enhancements of specific vibrational modes whose electronic absorptions align with 532 nm excitation.^8^ A potential source of this resonant enhancement are metal-binding proteins or metalloproteins, which can be excited by visible light ∼532 nm^21^ and have been widely implicated in NDDs including AD.^22^ In comparison to 785 nm fingerprints, the 532 nm spectra show enhanced signals for aromatic amino acids such as histidine and tryptophan and vibrations associated with the formation of metalloporphyrin (MP) structure. These include 747 cm^–1^ (Trp, indole ring), 880 cm^–1^ (Trp, H-bonding), 971 cm^–1^ (Trp), 1126 cm^–1^ (C–N), 1308 cm^–1^ (Trp), 1367 cm^–1^ (MP, C–N), 1587 cm^–1^ (MP, C–C), 1620 cm^–1^ (vinyl C=C).^23–25^ These vibrations are present but comparatively weak in the 785 nm fingerprint, which is dominated by protein backbone vibrations including peaks in the skeletal region (e.g., C–C, C–N, ∼880–1180 cm^–1^), the extended amide III region (predominantly N–H, C–N, ∼1200–1350 cm^–1^), CH_2_ deformation (∼1440 cm^–1^) and the amide I region (predominantly C=O, ∼1600–1700 cm^–1^), as well as some sharp side chain vibrations such as Phenylalanine (Phe, 1003 cm^–1^).^23^ We hypothesized that by combining 532 and 785 nm spectra into an MX-Raman fingerprint, we could maximize information content and increase the accuracy of NDD sample classification. We tested this with a simple abstraction process to integrate information using end-on-end concatenation of spectra.

**Figure 2:**
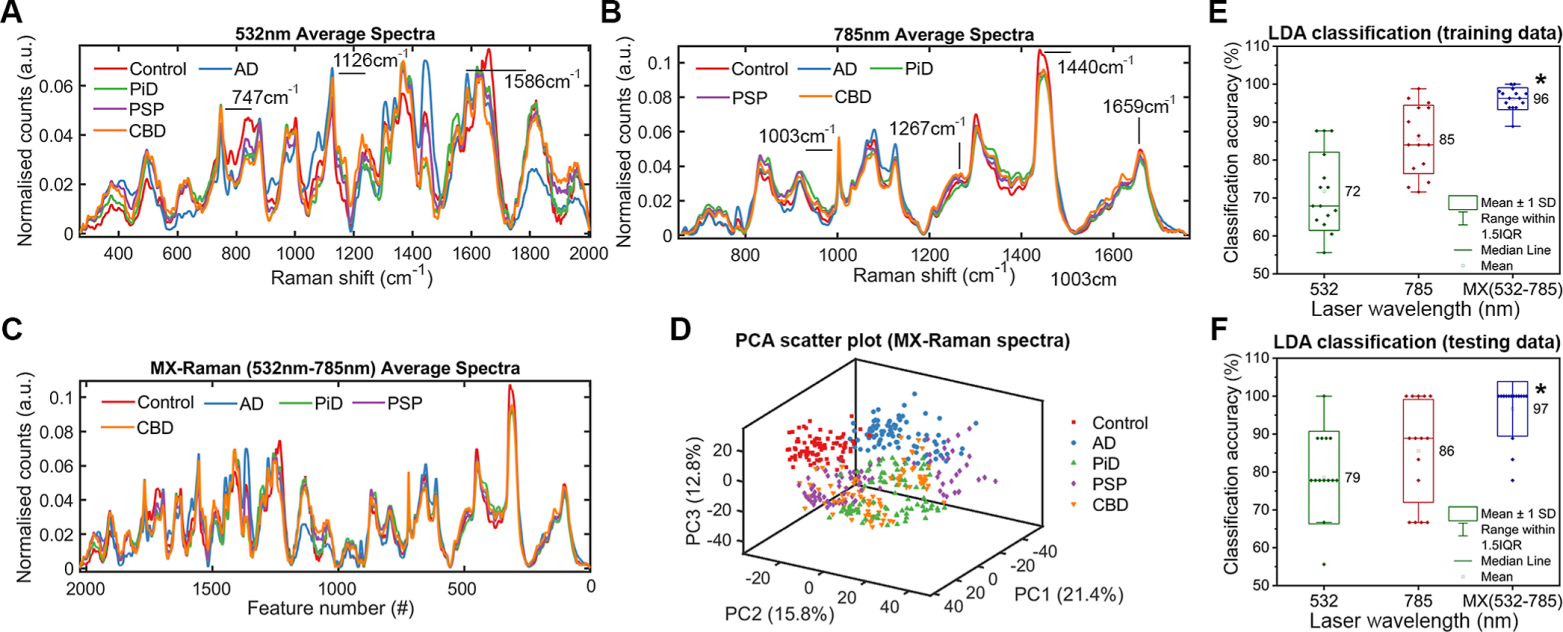
Wavelength-dependent MX-Raman improves classification accuracy. (A–C) Vector normalized, average Raman fingerprints for each class from 785 nm (A), 532 nm (B), and concatenated 532 nm–785 nm MX-Raman (C). Each of the fingerprints depicted is an average of 90 spectra, 30 from each sample (*N* = 15, *n* = 3 per disease class). (D) Scatter plot of all 450 532 nm-785 nm MX-Raman fingerprints reduced to 3 dimensions using PCA. (E–F) Box plots representing LDA classification of Raman and MX-Raman fingerprints with 5-fold cross validation. The LDA model was trained and optimized on 3 sets of training data (E) and evaluated on the 3 equiv sets of testing data (F). Each point represents the classification accuracy for each disease class (*N* = 15), * = *p* < 0.05.

The concatenated MX-Raman (532 nm–785 nm) fingerprints are shown in Figure 2C. PCA was applied to the MX-Raman (532 nm–785 nm) data set to transform the spectra into a set of orthogonal scores based on variance. The transformed spectra were projected onto a scatterplot consisting of the first 3 principal components (those which contain the majority of variance), representing ∼50% of the variance within the data set (Figure 2D). This resulted in clustering of the transformed spectra from the healthy control samples (red squares) away from the spectra of the NDD samples (AD, PiD, PSP and CBD). Some cluster overlap was seen for AD spectra (blue circles), while the spectra for the other diseases were not resolved into well-defined clusters, including PiD (green triangles), PSP (purple diamonds), and CBD (orange inverted triangles). As the intraclass variance was larger than the interclass variance (particularly for PiD, PSP and CBD) a supervised method was required to better classify the data.

We wanted to determine whether increased information content from MX-Raman improved the classification accuracy of the spectra from NDD tissue. We were limited by sample size (*N* = 15, *n* = 3) and could not build a reliable predictive model. Instead, we utilized LDA with 5-fold cross-validation as a classifier to systematically assess and compare MX-Raman configurations in the context of this sample set. To do this, we partitioned each spectral data set into 3 independent training and testing data sets at a 9:1 ratio. For each configuration, the data set contained 30 spectra for each of the 15 patients, resulting in 450 spectra, of which 405 were used for training and 45 for testing. Due to a limited number of samples, the first, middle and final 3 spectra from each sample were used as testing sets 1, 2 and 3, respectively, while the remaining spectra were retained for training data. While using correlated data in this way is not appropriate for constructing a predictive model for classification due to an overestimation of accuracy, it allowed us to directly compare Raman configurations and investigate whether additional spectral information was useful to better distinguish the classes in our spectral data set.

The average classification accuracies for the training and testing data sets are displayed for each wavelength configuration in Figure 2E,F, respectively. Using cross-validated LDA, 532 nm fingerprints were classified into 5 groups with an overall accuracy of 78.5% ± 12.2%, while a higher classification accuracy of 85.6% ± 13.2% was observed for 785 nm fingerprints. MX-Raman (532 nm–785 nm) significantly outperformed each of the wavelengths alone, with an average classification accuracy of 96.7% ± 7.2%. To control for the increased number of *x*-variables (or features) in the MX-Raman spectrum, we concatenated each of the 785 and 532 nm fingerprints to themselves, resulting in MX-Raman (532 nm-532 nm) and MX-Raman (785 nm-785 nm) fingerprints, each with 2016 variables. This did not result in improved classification in comparison to the 1013 variable individual spectral fingerprints, with average classification accuracies of 78.5% ± 12.2% and 79.3% ± 17.2% respectively (Table S1). Together, this demonstrates that improved classification accuracy from MX-Raman (532 nm-785 nm) is not reliant on increasing the absolute number of variables, but instead the addition of independent but descriptive variables. Spectra from each of the single-wavelength configurations used for Raman spectroscopy were classified with acceptable to high accuracies including for control (532 nm = 88.9%, 785 nm = 81.5%) and AD fingerprints (532 nm = 85.2%, 785 nm = 100%), with 785 nm PiD fingerprints also being well classified (96.3%). Raman spectra from PSP and CBD were classified with lower accuracy [532 nm; (PSP = 66.7%, CBD = 74.1%), 785 nm; (PSP = 72.2%, CBD = 77.8%)]. It is known that PSP and CBD have a high degree of clinical and pathological overlap^26^ and may therefore be difficult to distinguish, yet MX-Raman (532 nm–785 nm) spectra were classified with high accuracy in all cases [control = 100%, AD = 100%, PiD = 100%, PSP = 96.3%, CBD = 87.0%] unlike conventional (single-excitation) Raman spectroscopy (Table S1).

#### Increasing Information Content by Polarization Detection

Molecular symmetry affects the polarization of Raman scattered light and the depolarization ratio ρ = *I*_perpendicular_/*I*_parallel_, where a vibration that is totally symmetric is equal to ρ < 0.75. This means that the depolarization (ρ) spectrum can provide additional structural information about a molecule or mixture including the molecular orientation of functional groups, as demonstrated for insulin fibrils.^11^ Therefore, we hypothesized that samples from each of the different NDD tissues could have a distinct depolarization profile due in part to variations in the tau protein fibrils within each disease.^27^ Raman (ρ) fingerprints using 785 nm excitation are shown in Figure S1. Despite an increase in information content, combining conventional and polarized Raman (ρ) fingerprints did not significantly improve overall classification accuracy of NDD brain samples.

#### Increasing Information Content by Autofluorescence Detection

We hypothesized that different NDD samples could have different fluorescent profiles due to different molecular compositions. Autofluorescence was captured at the same time as Raman scattered light, and Raman information was removed by subtraction. Therefore, the autofluorescence spectrum contains the same number of variables as the Raman spectrum, albeit with less discernible features, specifically consisting of one broad peak in comparison to tens of sharper peaks in the Raman spectrum (Figure S2). Autofluorescence spectra alone could not be used to classify the NDD samples.

Despite increased information content, MX (Raman-fluor) did not improve classification accuracy in comparison to Raman alone, again demonstrating that the addition of independent but descriptive variables is essential for improved spectral classification. MX-Raman (532 nm-785 nm), was the standout choice to improve NDD sample classification.

### Spectral Barcoding

We have shown that increasing spectral information content using MX-Raman spectroscopy improves classification of NDD brain samples. Importantly, this effect is not caused by additional content alone, but by increased independent and complementary information, that is, more descriptive variables or features for classification. We next wanted to identify these variables for spectral and chemical assignment, and to engineer features for improved unsupervised clustering and classification of MX-Raman spectra.

Raman spectra have high dimensionality (in this case containing 1013 variables after preprocessing), with MX-Raman spectra containing twice that number (2016 variables). Much of this information may be noise or nondescriptive intraclass/intrasample variance, as well as correlated and redundant. The reduction of spectral dimensionality to include only those features that are descriptive of class can improve the accuracy of unsupervised clustering methods that are inherently unable to identify descriptive features. Supervised classification models also benefit from dimensionality reduction due to a decreased chance of overfitting to noise and a lower computational cost that together improve generalizability and therefore real-world application.^28^ Data reduction methods that rely on transformation such as PCA are unbiased and can be simply implemented but utilize the whole spectrum and can therefore retain intraclass variance that is unrelated to class, i.e. nondescriptive. This may be a problem for interpretability of classification from holistic measurement methods such as label-free spectroscopies that capture a lot of information.

To screen for descriptive features specific to each class, we performed a series of nonparametric statistical tests on the MX-Raman (532 nm–785 nm) fingerprints for the control class versus each disease class (see Materials and Methods). From each resulting test, we selected the 3 independent regions of the spectrum that ranked highest for significance. These regions were then combined to form an average MX-Raman “spectral barcode” for each disease, with black bars depicting a significant increase in signal intensity in disease spectra compared to control spectra, and gray depicting a significant decrease (Figure 3A). Increases and decreases in spectral barcode signal were consistent for each of the 3 patients in each class. Unsupervised statistical analysis (PCA) was used to drive clustering of the barcodes showing that class distinction is retained (Figure S3). To determine the impact on intraclass variance we utilized Mahalanobis distance analysis to quantify the spread of spectra within each cluster across the PC1 and PC2 space (Figure 3B). This measure acknowledges that clusters are not necessarily spherical and instead measures the distance from each point to the center of mass of the cluster, divided by the cluster ellipsoid width in that direction.^29^ The Mahalanobis distance for each cluster is decreased when using the disease-specific spectral barcode as opposed to the whole MX-Raman spectrum for PCA analysis. As each cluster is in a PC space, this means that there is less intraclass variance for each spectral barcode in comparison to the whole MX-Raman spectrum, while class distinction is retained. Therefore, spectral barcodes contain the descriptive variables that are specific to each class while variables corresponding to intraclass noise are removed.

**Figure 3:**
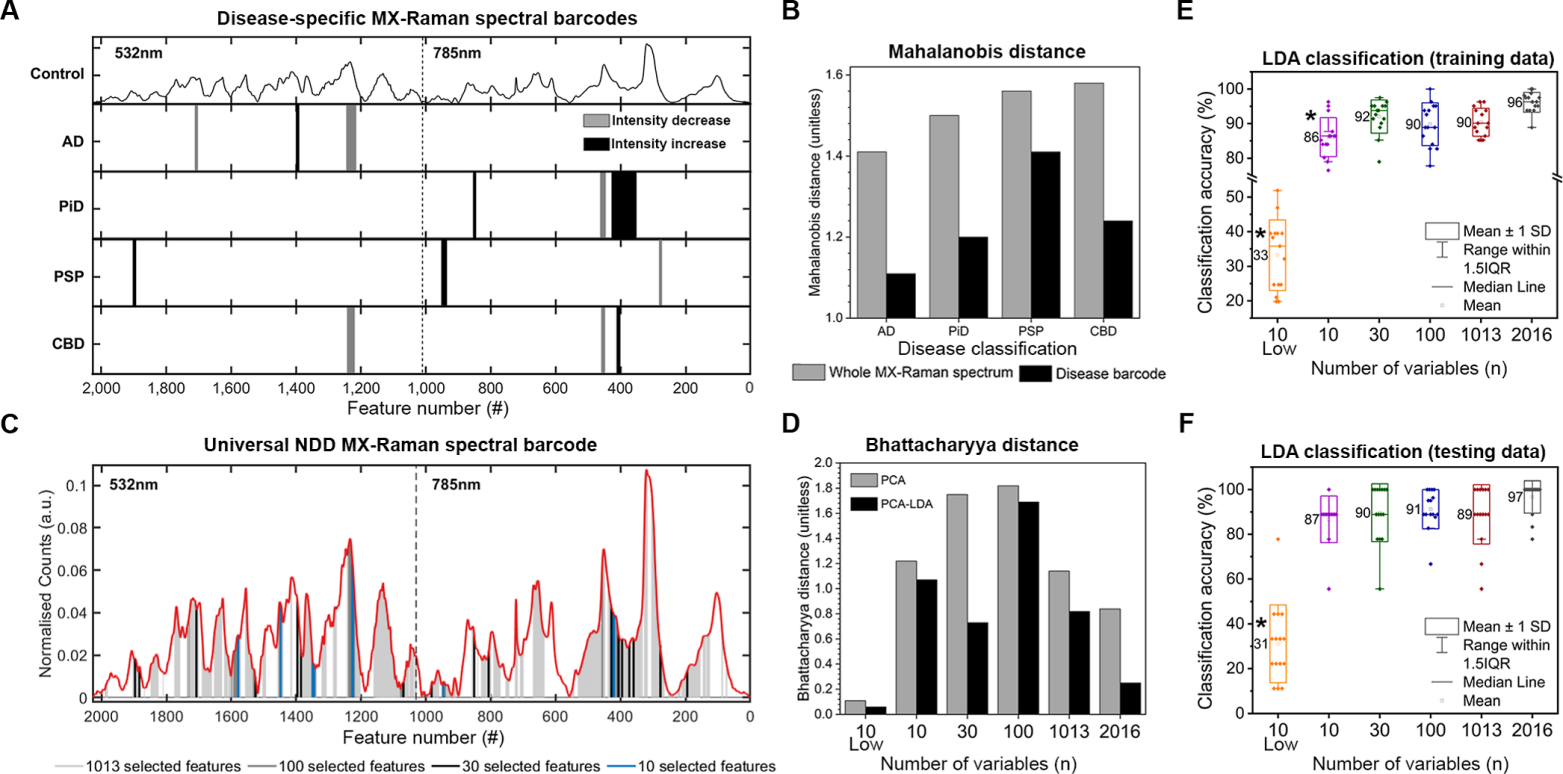
Intelligent Feature engineering for MX-Raman spectral barcodes. (A) Features representing spectral regions selected from MX-Raman fingerprints to generate disease-specific barcodes. A significant increase in signal for disease classes is indicated by black bars and a significant decrease in signal for disease cases is indicated by gray bars. (B) Bar chart showing Mahalanobis distance analysis of PCA transformed spectra for each disease cluster using the whole MX-Raman spectrum (gray) and the disease-specific MX-Raman spectral barcodes (black). (C) MX-Raman spectral barcodes were engineered with 1013 features (light gray), 100 features (dark gray), 30 features (black), and ten features (blue). (D) Bar chart showing average Bhattacharyya distance analysis for clusters from PCA (gray) and PCA-LDA (black) transformed MX-Raman barcodes. The first 3 PCs were retained for LDA. (E,F) Box plots representing LDA classification of MX-Raman fingerprints with 5-fold cross validation. The LDA model was trained and optimized on 3 sets of training data (E) and evaluated on the 3 equiv sets of testing data (F). Each point represents the classification accuracy for each disease class (*N* = 15), * = *p* < 0.05.

The key spectral differences between the non-NDD control samples and each disease class is captured by the disease-specific spectral barcodes. Importantly, the whole range of the MX-Raman spectrum is represented in these spectral barcodes with comparable contribution from each of the 532 and 785 nm components. Features in the AD spectral barcode are comprised entirely from variables in the 532 nm region, while features in the PiD spectral barcode are made up only of variables from the 785 nm region. Features in the PSP and CBD spectral barcodes are a combination of variables from across the complete MX-Raman spectral range. This confirms that the tissue fraction from each disease consists of a unique multicomponent mixture with a distinct Raman cross-section arising from differential sensitivity to each of the excitation wavelengths.

We next wanted to create a single MX-Raman spectral barcode that could be used for unsupervised clustering and classification of all the MX-Raman spectra. We also wanted to determine the optimal number of variables to include in this spectral barcode. We constructed 4 spectral barcodes of decreasing variable size (Figure 3C); retaining 1013 variables (light gray), 100 variables (dark gray), 30 variables (black) and 10 variables (blue). Of the 10 variables in the minimal MX-Raman spectral barcode, 6 arise from the 532 nm fingerprint and 4 from the 785 nm fingerprint (Table S2). Vibrations from nonregular/β-sheet conformations best explain the difference between control and AD spectra, as well as control and CBD spectra, possibly arising from aggregated protein fibrils in the insoluble tissue fractions of disease cases. Tau variably makes up ∼20% of the total protein in the insoluble fraction for AD brain tissue and is therefore likely to make a large contribution to the Raman spectrum.^30^ To validate this, we performed SDS-PAGE to separate the proteins in the insoluble fraction of each brain sample followed by antitau Western Blot analysis to visualize the aggregated tau protein specifically. Qualitatively, we identified higher levels of tau protein in the insoluble fraction of AD and CBD brains in comparison to those of PiD and PSP (Figure S4). Tryptophan (Trp) and histidine (His) side chain vibrations corresponding to metalloporphyrin (MP) structure were also well represented in the minimal spectral barcode, with histidine being most associated with heme group formation, with contributions also observed from aromatic amino acids.^31^ Vibrations from the protein-backbone and RNA are also important to distinguish MX-Raman spectra from PiD, PSP and CBD. RNA-binding proteins have been identified in insoluble brain fractions from AD cases so may also be found in other NDDs.^32^

#### MX-Raman Spectral Barcode Classification

To assess the optimal number of features for unsupervised clustering of MX-Raman spectra, we used PCA followed by Bhattacharyya distance analysis to determine the overlap between the ellipsoids of each class.^33^ The average Bhattacharyya distance for the PCA clusters from each of the MX-Raman spectral barcodes is shown in Figure 3D (gray bars). Bhattacharyya distance for the PCA clusters from the whole MX-Raman spectrum (2016 variables = 0.84) is lower than that for each of the spectral barcodes (1013 variables = 1.14, 100 variables = 1.82, 30 variables = 1.75, 10 variables = 1.22), meaning cluster overlap is reduced after feature engineering. PCA scatter plots depicting the clusters are shown in Figure S5. Of the 4 spectral barcodes tested, the optimal number of variables for PCA clustering was 100, as the transformed spectral barcode had the largest average Bhattacharyya distance measurement and therefore the lowest amount of cluster overlap. As a negative control, we selected the 10 lowest ranking features using analysis of variance (ANOVA) and repeated the Bhattacharyya distance analysis. We observed a reduction in Bhattacharyya distance (10 lowest ranked variables = 0.11) and therefore an increase in cluster overlap. This further confirms that the independence and complementarity of the information provided by each feature is more important than the absolute number of variables.

Classification of MX-Raman spectral barcodes in each cluster was achieved using cross-validated LDA of the 3 PCs and the Bhattacharyya distance was calculated for the resulting clusters (Figure 3D, black bars). A similar pattern was observed for the measurements of Bhattacharyya distance for each MX-Raman spectral barcode transformed using PCA and cross-validated PCA-LDA, with the PCA transformation alone leading to less cluster overlap than LDA which is optimized to maximize the separation of means as opposed to individual data points. Importantly, each of the spectral barcodes were classified with higher Bhattacharyya distance and therefore less cluster overlap by PCA-LDA (1013 variables = 0.82, 100 variables = 1.69, 30 variables = 0.73, 10 variables = 1.07) than the whole MX-Raman spectrum (2016 variables = 0.25). Clustering of classes was not observed for the 10 lowest ranked variables identified using ANOVA (10 variables = 0.06). PCA-LDA scatter plots depicting the clusters are shown in Figure S5. In all cases, clustering was lost when class labels were randomly shuffled, ruling out overfitting of the cross-validated PCA-LDA model. This confirms that intraclass variance is reduced through feature engineering of MX-Raman spectral barcodes while interclass variance is retained, and now the major source of variance.

We next wanted to compare the accuracy of classification using each MX-Raman spectral barcode. To do this, we utilized the original training and testing data sets analyzed by direct LDA in Figure 2. MX-Raman Spectra were reduced from 2016 variables to 1013, 100, 30, 10 highest ranked and 10 lowest ranked variables, and for each case the LDA model was retrained. We compared the overall classification accuracy using each MX-Raman spectral barcode to that using the complete MX-Raman spectral range. A significant decrease in classification accuracy was observed for the MX-Raman barcode consisting of the 10 lowest ranked variables for the training data (33.2% ± 10.2%, Figure 3E) and testing data (31.1% ± 17.4%, Figure 3F), underlining the importance of feature specificity. For the training data, we also observed a significant decrease in classification accuracy for the 10 highest ranked variable barcode resulting in 86.1% ± 5.6% classification accuracy (Figure 3E). We did not observe any significant reduction in classification accuracy for the MX-Raman barcodes consisting of 30, 100, or 1013 variables (Table S3). Together, this shows that spectral features from each wavelength provide uniquely descriptive information, and that the 10 features in the minimal spectral barcode encode a majority of the disease-specific information in the MX-Raman spectrum that is necessary for classification of NDD brain samples. Based on the analyses presented, retaining 100 variables enables MX-Raman spectra of NDD brain samples to be clustered and classified most accurately.

## Discussion

In this study we observed that increasing information content through MX-Raman improved the classification of NDD samples, but only when that extra information was independent and descriptive. While we observed that combining different polarization configurations enhanced the overall classification accuracy of spectra from NDD samples by ∼5%, this difference was not significant while the combination of two distinct laser wavelengths, specifically 532 and 785 nm, significantly increased classification accuracy by >10%. This effect results from the differential resonant and preresonant enhancement of vibrational modes in each of the multicomponent samples.^8^ A limitation of this proof-of-concept study was sample size (*N* = 15, *n* = 3), so any conclusions related to NDD classification are thus caveated accordingly as we could not robustly develop a predictive model and our analyses were implemented for the comparison of Raman and MX-Raman methodologies. Larger cross-sectional studies are required to further validate NDD classification using MX-Raman. Despite this, our analyses have demonstrated that increasing spectral information content using MX-Raman fundamentally improves the discrimination of Raman fingerprints derived from different complex mixtures. Importantly, discrimination was only improved when complementary Raman information was added using distinct wavelengths and not when variables were duplicated computationally, or through collecting non-Raman (autofluorescence) information with an equal number of variables.

Raman spectroscopy is routinely performed using a range of laser wavelengths typically in the visible spectrum. Matching at least one of these laser wavelengths with preresonance/resonance of components in the sample can maximize the content of spectral information for MX-Raman. Here, we observed the preresonant/resonant enhancement of metalloproteins using 532 nm excitation, while a 785 nm excitation did not cause any observable enhancement. The role of metal imbalance in NDD progression has been substantially reviewed,^34^ particularly for AD,^22^ with metal ions including iron, copper, zinc, magnesium and manganese implicated. Metal ions, and metalloproteins in particular, may be useful biomarkers for NDD onset and progression.^35^ Ferritin is a major store for brain iron and is also a major component of the insoluble proteome of the brain.^36^ The absorption window for ferritin (∼550 nm) that enables the resonant enhancement of Raman bands,^21^ is shared with hemoglobin and cytochrome C,^37^ and metal complex fragments of tau^38^ and amyloid-β,^39^ suggesting that the 532 nm Raman fingerprint represents multiple insoluble brain metalloproteins.

While distinct wavelengths could be combined into MX-Raman fingerprints to improve NDD classification accuracy, different polarization states and the autofluorescence signatures that are normally removed during Raman preprocessing encoded less disease-specific information. While autofluorescence is measured together on the same detector as Raman scattered photons, it must also be noted that the instrumentation requirements for polarized Raman (filters) are relatively simpler and inexpensive compared to the incorporation of a second laser source into a Raman spectrometer system. This may be a particularly important trade-off when considering clinical translation of MX-Raman methodologies for different applications.

The insoluble fraction from NDD brain samples investigated in this study has also been analyzed using mass spectrometry (MS) to identify AD-associated proteins that corelate with disease onset and progression,^40^ and to identify proteins that are not present in other NDDs such as frontotemporal lobar degeneration (FTLD).^41^ It has also been shown that MS signatures of the insoluble proteome from AD brain and other NDDs are distinct from that of control patients and from each other.^42^ Translational research has also been performed to demonstrate that MS is an effective tool for biomarker discovery in CSF from heterogeneous AD cohorts,^43^ as well as from blood plasma at early stages of AD.^44^ While MS excels at molecular identification and sample classification, MX-Raman has the edge in terms of simplicity, and potential for miniaturization and portability and thus affordability and scalability. This gives Raman-based approaches an advantage in real-world clinical deployment, but they are yet to be robustly validated in patient biofluids beyond promising proof-of-concept studies.^17,45^

Our present work provides proof-of-concept that MX-Raman spectroscopy can be used to detect and distinguish complex biological samples, specifically pathological brain fractions from a range of clinically overlapping NDDs. These samples are taken from defined regions of the brain, known to be especially affected, and further processed to enhance any detectable differences between each disease. It is important to note that the concentrations of pathological protein present in patient biofluid are orders of magnitude lower by comparison. Nevertheless, Raman spectra are not reliant on only a specific analyte such as tau or ferritin but instead capture the overall composition of a sample and can therefore detect global molecular changes. Descriptive information content can be increased by combining holistic Raman spectroscopy with more specific preresonance/resonance or polarized Raman spectroscopy, and redundant features can be removed using our spectral barcoding approach, which together can be translated and applied to any classification problem in a broad range of disciplines including NDD diagnostics and beyond.

## Materials and Methods

### Brain Samples

300 mg of tissue from the cerebral cortex was used for experiments. Control and AD brain tissues were sourced from the South West Dementia Brain Bank (Bristol, UK) and primary Tauopathy brain tissues (PiD, PSP and CBD) were sourced from the Brains for Dementia Research, London Neurodegenerative Disease Brain Bank (London, UK). Details about tissue donors are shown in Table S4.

### Tissue Homogenization and Preparation

All reagents were purchased from Merck unless otherwise stated. 300 mg brain tissue was added to a 5 mL borosilicate homogenizer (Fisherbrand) and 5× (1.5 mL) ice cold A68 buffer (10 mM Tris–HCl pH7.4, 800 mM NaCl, 1 mM EGTA, 10% sucrose, 1× cOmplete, EDTA-free Protease Inhibitor Cocktail) was added and tissue was homogenized by 30 up–down mortar strokes on ice. Tissue was centrifuged at 20,000*g* for 20 min at 4 °C and the pellet was discarded. Remaining supernatant was combined at a 1:4 ratio with insoluble preparation buffer (10 mM Tris–HCl pH7.4, 800 mM NaCl, 1 mM EGTA and 10% sucrose, 4% *N*-Lauryl sarcosine sodium salt, 1× cOmplete, EDTA-free Protease Inhibitor Cocktail) and incubated for 1 h with rotation at RT. Samples were centrifuged at 150,000*g* for 1 h at 4 °C and supernatant was removed. Pellets were then washed three times in H_2_O and finally resuspended in 2 μL H_2_O. After mixing well with a pipet, 0.25 μL of this sample was deposited by drop-deposition onto a hydrophobic surface treated 0.5 mm fused quartz coverslip (UQG Optics) and dried in a vacuum chamber before same-day Raman analysis as described previously.^6^ Three samples were measured each day in a blinded fashion, with each of the 3 samples from a different class to avoid experimenter and instrument bias, respectively.

### Raman Spectroscopy

A Renishaw inViaTM Qontor microscope system was used for Raman spectroscopy. Data was collected and parameters were determined using Renishaw WIRE5.5 software. The Raman system was calibrated to the 520–521 cm^–1^ reference peak of the internal silicon substrate prior to each experiment. The charge-coupled device (CCD) detector and spectrometer slit areas were aligned using the auto align function and the laser spot was manually aligned to the center of the crosshairs using the camera. Dried droplets were located and brought into focus using a Leica DM 2500 M bright field microscope and an automated 100 nm-encoded *XYZ* stage. For Raman spectroscopy, 30 spectra were collected from roughly evenly spaced locations around the center of the outer “coffee ring”. The samples were excited using a 532 nm laser at 10% power (0.17 mW at sample) or a 785 nm laser at 100% power (11.66 mW at sample) focused through a Leica 100× short working distance objective (numerical aperture = 0.85). Background quartz spectra were measured in 3 roughly evenly spaced locations around the dried droplet using equivalent *Z* distances as for each sample measurement. As multiple acquisitions were acquired per spectrum, cosmic rays were removed manually after each spectral measurement.

### Raman Spectral Preprocessing and Feature Selection

Preprocessing and feature selection was performed using the IRootLab plugin (0.15.07.09-v) for MATLAB R2023a.^46^ All spectra were background-subtracted using blank quartz spectra and high-frequency noise was removed using the Haar-wavelet denoising function with 6 decomposition levels. A fifth-order polynomial was used to remove fluorescence, and the ends of each spectrum were anchored to the axis using the rubberband-like function. Spectral intensity normalization was applied using vector normalization and spectra were standardized for PCA.

For feature selection, nonparametric U-tests were run per wavenumber in a pairwise manner for each class. For disease barcodes, 4 *U*-tests were performed, one for each class against the control class. The 3 highest ranking variables in independent spectral regions were selected and the entirety of each of these 3 spectral regions were retained. In this case, a spectral region corresponded to an unbroken sequence of variables that was independent of the overall number of variables in that region. For the universal NDD barcode, 10 U-tests were performed, one between each of the 5 classes. The 10 highest ranking variables were selected, independent of region, and reduced to 3 variables using a minimum redundancy maximum relevance (mRMR) algorithm (MATLAB; fscmrmr). These 3 variables from each *U*-test were used to make the 30-variable NDD barcode, while the highest mRMR ranking variable from each *U*-test was used to make the 10-variable mRMR barcode.

### Statistical and Multivariate Analysis

Principal component analysis (PCA) was performed using the IRootLab plugin (0.15.07.09-v) for MATLAB R2023a^46^ and three PCs were retained for depiction of transformed data in scatter plots. For PCA-linear discriminant analysis (LDA), The first 3 PCs were retained before LDA which resulted in 4 LDs. The first 3 LDs were retained for depiction of the transformed spectra in scatter plots.

LDA classification and spectral prediction was performed using the MATLAB Classification Learner application. Data was first split into training (90%) and testing (10%) sets at the level of spectrum. LDA was trained on the training data sets using 5-fold cross validation before testing for classification of the data into 5 distinct groups (control, AD, PiD, PSP and CBD). Training and testing was repeated 3 times using the first, middle, and final spectra from the spectral data set of each sample. Results are displayed as an average and the standard deviation of the mean across the resulting 15 values. The nonparametric Kruskal–Wallis test with Bonferroni correction was used to compare the classification accuracies obtained for each laser configuration, with two-sided *P* < 0.05 considered statistically significant.

## Supplementary Material



## Notes

Additional experimental details, materials, and methods, tables and figures, including polarized Raman and autofluorescence measurements, and Western blot analysis (PDF)
