# Supplementary material for: A Novel Spectral Barcoding and Classification Approach for Complex Biological Samples Using Multiexcitation Raman Spectroscopy (MX-Raman)
Source: Anal Chem. 2025 Jun 3;97(23):12189–97. doi: 10.1021/acs.analchem.5c00776 (PMC12177875; doi:10.1021/acs.analchem.5c00776)
Supplement: Supplementary file 1 [file ac5c00776_si_001.pdf]

Supporting Information

A Novel Spectral Barcoding and Classification Approach for Complex Biological Samples using Multi-excitation Raman Spectroscopy (MX-Raman)

George Devitt[‡§‖]*, Niall Hanrahan[§‖], Miguel Ramírez Moreno[‡‖], Amrit Mudher[‡‖] and Sumeet Mahajan[§‖]*

‡School of Biological Sciences, University of Southampton, Highfield Campus, SO17 1BJ, Southampton, UK

§School of Chemistry and Chemical Engineering, University of Southampton, Highfield Campus, SO17 1BJ, Southampton, UK

‖Institute for Life Sciences, University of Southampton, Highfield Campus, SO17 1BJ, Southampton, UK

Table of Contents

Table S1. Average classification accuracy of wavelength-dependent MX-Raman using direct LDA S2
Table S2. Highest ranking variable from each feature selection S3
Table S3. Average classification accuracy of MX-Raman barcodes using direct LDA S4
Table S4. Tissue donor information S5

Figure S1. Polarization-dependent MX-Raman does not improve classification accuracy S6
Figure S2. Autofluorescence-dependent MX-Raman does not improve classification accuracy................. S7
Figure S3**.** Disease barcodes reduce intraclass variance. S8
Figure S4. Tauopathy subtype determines insoluble tau protein concentration....................................... S9
Figure S5. 100 features are optimal for clustering of MX-Raman barcodes S10

Detailed experimental methods S11

References.. S13

Table S1. Average classification accuracy of wavelength-dependent MX-Raman using direct LDA										
	**532nm**		**532nm-532nm**		**785nm**		**785nm-785nm**		**532nm-785nm**	
Test set	Train	Test	Train	Test	Train	Test	Train	Test	Train	Test
Control	74.9%	88.9%	81.5%	88.6%	84.8%	81.5%	85.6%	74.1%	97.5%	100.0%
AD	87.7%	85.2%	85.6%	85.2%	96.3%	100.0%	95.1%	96.3%	99.6%	100.0%
PiD	65.0%	77.8%	65.9%	77.8%	93.0%	96.3%	89.7%	92.6%	95.5%	100.0%
PSP	60.1%	66.7%	58.4%	66.7%	74.1%	72.2%	74.5%	63.0%	95.2%	96.3%
CBD	71.2%	74.1%	69.6%	74.1%	79.0%	77.8%	77.4%	70.4%	93.0%	87.0%
**Average**	**71.8%** (±10.3%)	**78.5%** (±12.2%)	**72.2%** (±10.6%)	**78.5%** (±12.2%)	**85.4%** (±9.0%)	**85.6%** (±13.6%)	**84.5%** (±8.4%)	**79.3%** (±17.2%)	**96.2%*** (±2.9%)	**96.7%*** (±7.2%)
Adjusted P values representing significant differences in average classification accuracy indicated with an asterisk (* = P < 0.05)										

Table S2. Highest ranking variable from each feature selection					
Group	Feature no. (a.u.)	Laser wavelength (nm)	Raman shift (cm^{-1})	Nearest peak (cm^{-1})	Assignment
Control vs AD	1224	532	1672	1661	Protein amide I nonregular/β-Sheet
Control vs PiD	372	785	1385	1396	COO stretch/RNA
Control vs PSP	947	785	740	743	Trp, indole ring
Control vs CBD	1226	532	1675	1661	Amide I nonregular/β-sheet
AD vs PiD	1349	532	1473	1483	His/MP
AD vs PSP	1578	532	1087	1080	Phe/MP
AD vs CBD	1346	532	1478	1483	His/MP
PiD vs PSP	421	785	1335	1340	C_a-H def/ Trp/RNA
PiD vs CBD	1448	532	1308	1310	Trp/His/MP
PSP vs CBD	420	785	1334	1340	Ca-H def/ Trp/RNA
Peak assignments (1-6)					

Table S3. Average classification accuracy of MX-Raman barcodes using direct LDA

	**All 2016 features**		**1013 highest ranked features**		**100 highest ranked features**		**30 highest ranked features**		**10 highest ranked features**		**10 lowest ranked features**	
Test set	Train	Test	Train	Test	Train	Test	Train	Test	Train	Test	Train	Test
Control	97.5%	100.0%	90.9%	100%	86.4%	92.2%	90.1%	88.9%	84.8%	85.2%	39.1%	29.6%
AD	99.6%	100.0%	95.5%	88.9%	92.6%	94.7%	94.7%	96.3%	95.1%	92.6%	46.1%	55.5%
PiD	95.5%	100.0%	91.0%	81.5%	89.3%	95.1%	96.3%	96.3%	85.6%	92.6%	21.8%	14.8%
PSP	95.2%	96.3%	86.8%	88.9%	95.9%	94.7%	82.3%	70.4%	78.6%	74.1%	35.8%	33.3%
CBD	93.0%	87.0%	87.7%	85.2%	84.8%	79.4%	95.1%	92.6%	86.4%	88.9%	23.1%	22.2%
**Average**	**96.3%** (±2.9%)	**97.0%** (±7.2%)	**91.4%**$^{N.S}$ (±4.0%)	**88.9%**$^{N.S}$ (±13.3%)	**90.6%**$^{N.S}$ (±6.2%)	**90.4%**$^{N.S}$ (±8.8%)	**91.8%**$^{N.S}$ (±4.8%)	**88.9%**$^{N.S}$ (±12.9%)	**86.1%*** (±5.6%)	**86.7%**$^{N.S}$ (±10.4%)	**33.2%*** (±10.2%)	**31.1%*** (±17.4%)

Adjusted P values representing significant differences in average classification accuracy indicated with an asterisk (* = P < 0.05)

Table S4. Tissue donor information				
Patient code	Sex	Age	PM delay (hours	Pathology diagnosis
PiD1	M	61	23	Pick's disease
PiD2	M	72	6	Pick's disease
PiD3	F	67	38.5	Pick's disease
PSP1	M	67	55	Progressive supranuclear palsy
PSP2	M	84	22	Progressive Supranuclear Palsy
PSP3	F	84	9	Progressive Supranuclear Palsy
CBD1	M	73	35.5	Corticobasal Degeneration
CBD2	M	70	6.5	Corticobasal Degeneration
CBD3	F	73	25	Corticobasal degeneration
C1	M	78	51.5	Control
C2	F	70	33.25	Control
C3	F	89	26.5	Control
AD1	F	88	7.5	Alzheimer's disease
AD2	M	77	21.75	Alzheimer's disease
AD3	F	90	21.25	Alzheimer's disease

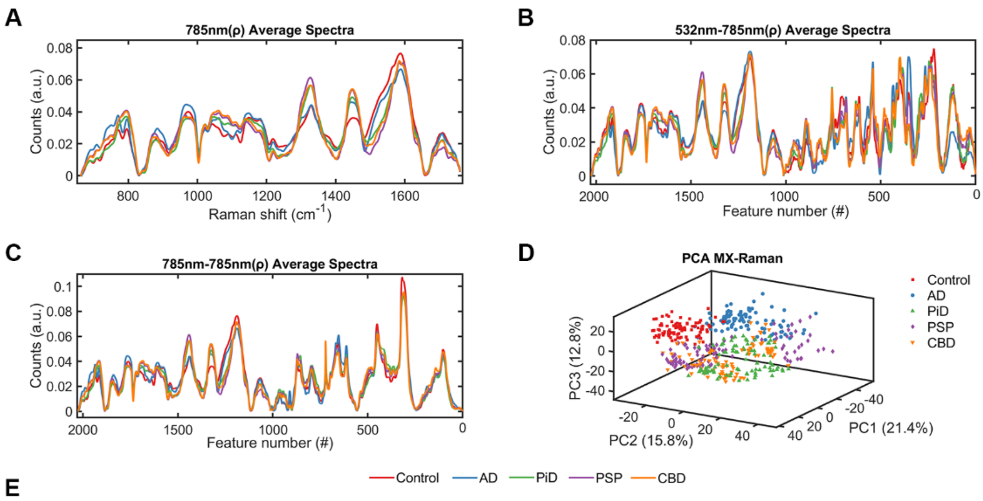

E

Average classification accuracy using direct LDA (5-fold cross-validation)										
	532nm		785nm		785nm(ρ)		532nm-785nm(ρ)		785nm-785nm(ρ)	
Test set	Train	Test	Train	Test	Train	Test	Train	Test	Train	Test
Control	74.9%	88.9%	84.8%	81.5%	95.4%	96.3%	93.0%	92.6%	95.5%	96.3%
AD	87.7%	85.2%	96.3%	100.0%	86.0%	85.2%	98.8%	96.3%	97.5%	88.9%
PiD	65.0%	77.8%	93.0%	96.3%	78.2%	70.4%	94.2%	96.3%	96.3%	92.6%
PSP	60.2%	66.7%	74.1%	72.2%	68.3%	63.0%	79.8%	66.7%	88.5%	88.9%
CBD	71.2%	74.1%	79.0%	77.8%	75.3%	77.8%	88.1%	85.2%	92.2%	88.9%
**Average**	**73.7%** (±10.3%)	**78.5%** (±12.2%)	**85.5%** (±9.0%)	**85.9%** (±13.6%)	**80.6%** (±17.2%)	**78.5%** (±10.1%)	**90.4%**$^{N.S}$ (±6.8%)	**87.2%**$^{N.S}$ (±12.5%)	**94.0%**$^{N.S}$ (±3.7%)	**91.1%**$^{N.S}$ (±7.5%)
Adjusted P values representing significant differences in average classification accuracy indicated with an asterisk (N.S = P > 0.05)										

Figure S1. Polarization-dependent MX-Raman does not improve classification accuracy

A-C. Vector normalized, average Raman fingerprints for each class including 785nm depolarization (ρ) (A), concatenated 532nm-785nm(ρ) MX-Raman (B), and concatenated 785nm-785nm(ρ) MX-Raman (C). Each of the fingerprints depicted is an average of 90 spectra, 30 from each patient. D. PCA analysis of the 785nm-785nm(ρ) MX-Raman fingerprints. Each point represents one of thirty spatially independent spectra from 3 independent samples per class. E. LDA classification with 5-fold cross validation. The LDA model was retrained and tested 3 times. Average and standard deviation calculated from the 15 average values for each wavelength combination.

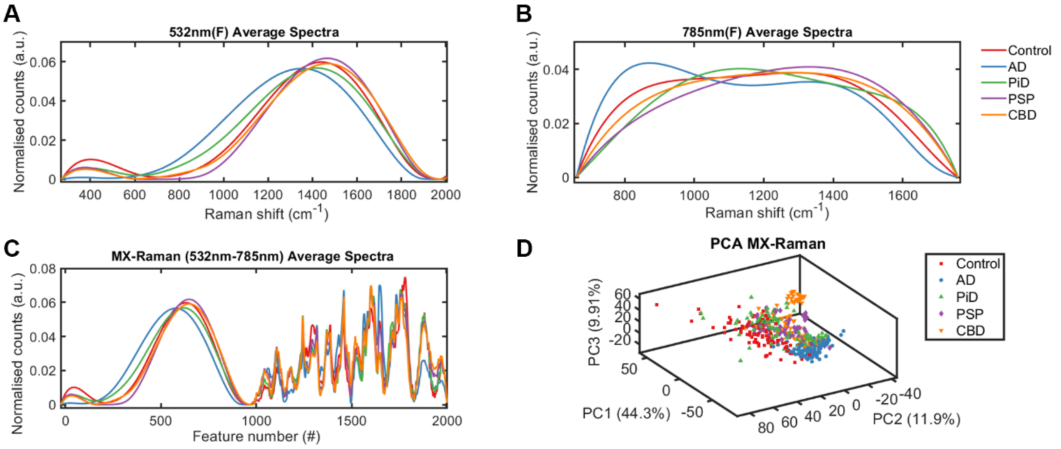

E

Average classification accuracy using direct LDA (5-fold cross-validation)										
	532nm		532nm-532nm(F)		785nm		785nm-785nm(F)		532nm-785nm	
Test set	Train	Test	Train	Test	Train	Test	Train	Test	Train	Test
Control	74.9%	88.9%	90.1%	85.2%	84.8%	81.5%	93.0%	92.6%	97.5%	100.0%
AD	87.7%	85.2%	72.4%	70.4%	96.3%	100.0%	98.8%	96.3%	99.6%	100.0%
PiD	65.0%	77.8%	68.3%	70.4%	93.0%	96.3%	94.2%	96.3%	95.5%	100.0%
PSP	60.2%	66.7%	74.1%	88.9%	74.1%	72.2%	79.8%	66.7%	95.2%	96.3%
CBD	71.2%	74.1%	81.5%	88.9%	79.0%	77.8%	88.1%	85.2%	93.0%	87.0%
**Average**	**73.7%** (±10.3%)	**78.5%** (±12.2%)	**77.3%**$^{N.S}$ (±8.9%)	**80.8%**$^{N.S}$ (±12.9%)	**85.5%** (±9.0%)	**85.9%** (±13.6%)	**88.7%**$^{N.S}$ (±5.9%)	**85.9%**$^{N.S}$ (±14.2%)	**96.2%*** (±2.9%)	**96.7%*** (±7.2%)
Adjusted P values representing significant differences in average classification accuracy indicated with an asterisk (N.S = P > 0.05)										

Figure S2. Autofluorescence-dependent MX-Raman does not improve classification accuracy.

A. Average 532nm autofluorescence (F) fingerprints for each disease group. **B.** Average 785nm autofluorescence (F) fingerprints for each disease group. **C.** Average 532nm-532nm(F) MX-Raman fingerprints for each disease group created by appending each of the independently measured 532nm and 532nm(F) Raman fingerprints end-to-end. **D.** Scatterplot depicting the spectral variance across the PC1, PC2, and PC3 axes from PCA analysis of the 532nm-532nm(F) MX-Raman fingerprints. **E.** LDA classification with k-folds cross validation (5-folds) was performed 3 times for each wavelength combination using either the first, middle, or final 3 spectra for each sample in the testing group. Average and standard deviation calculated from the 15 average values for each wavelength combination.

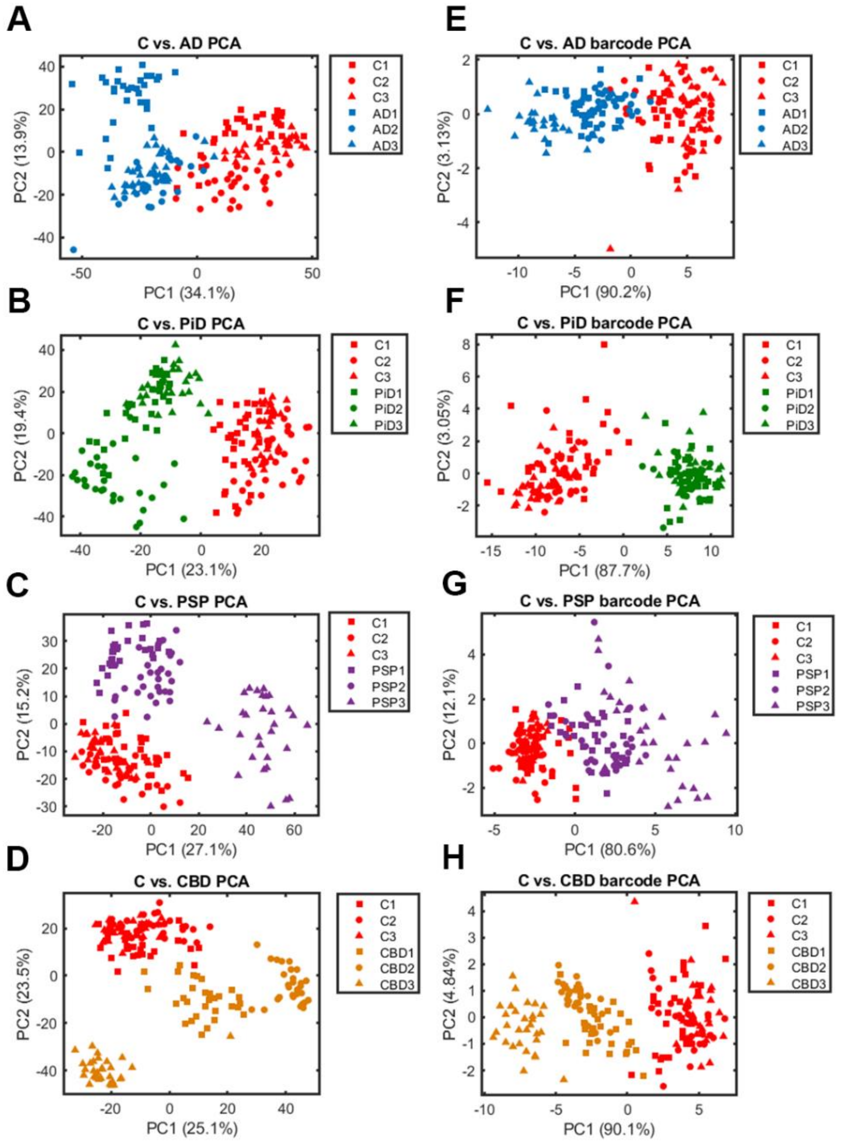

Figure S3**.** Disease barcodes reduce intraclass variance.

Pairwise PCA of MX-Raman (532nm-785nm) spectra from healthy control samples versus spectra from each disease case depicted as scatter plots retaining PC1 and PC2 including; AD (A and E), PiD (B and F), PSP (C and G), CBD (D and H). Scatter plots in the left-hand column depict PCA utilising the full MX-Raman (532nm-785nm) spectrum. Scatter plots in the right-hand column depict PCA utilising the disease-specific Raman barcode. Diseases are depicted as described in each title and key. Each individual sample was also assigned a different shape to highlight intra-class variability. The PCA scatter plots for each disease-specific Raman barcode shows a reduction in inter-sample variance within each class whilst disease/class relevant descriptive variance is retained. This data was used for Mahalanobis distance analysis shown in Figure 3B of the main manuscript.

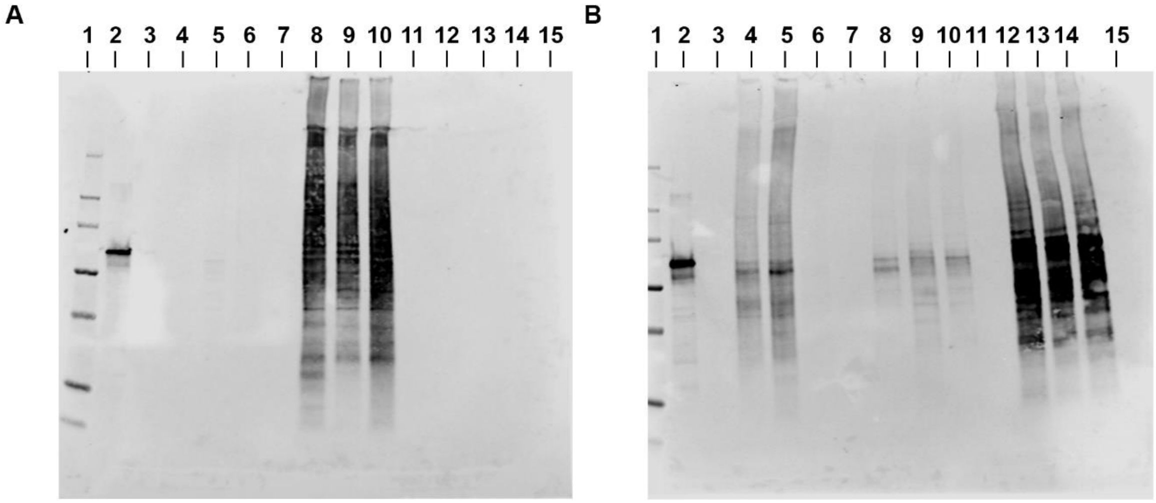

Figure S4. Tauopathy subtype determines insoluble tau protein concentration

SDS-PAGE and western blot analysis of insoluble brain fraction from **A.** Control (C) and AD: 1 = reference protein ladder, 2 = recombinant tau protein, 3 = blank, 4 = C1, 5 = C2, 6 = C3, 7 = blank, 8 = AD1, 9 = AD2, 10 = AD3, 11-15 = blank. **B.** Primary tauopathies; Pick's disease, PSP, and CBD (left to right). 1 = reference protein ladder, 2 = recombinant tau protein, 3 = blank, 4 = PiD1, 5 = PiD2, 6 = PiD3*, 7 = blank, 8 = PSP1, 9 = PSP2, 10 = PSP3, 11 = blank, 12 = CBD1, 13 = CBD2, 14 = CBD3, 15 = blank. Protein ladder and purified recombinant tau were used as a molecular weight references. * Note: PiD3 does not show a tau signal due to loss of insoluble pellet during sample processing.

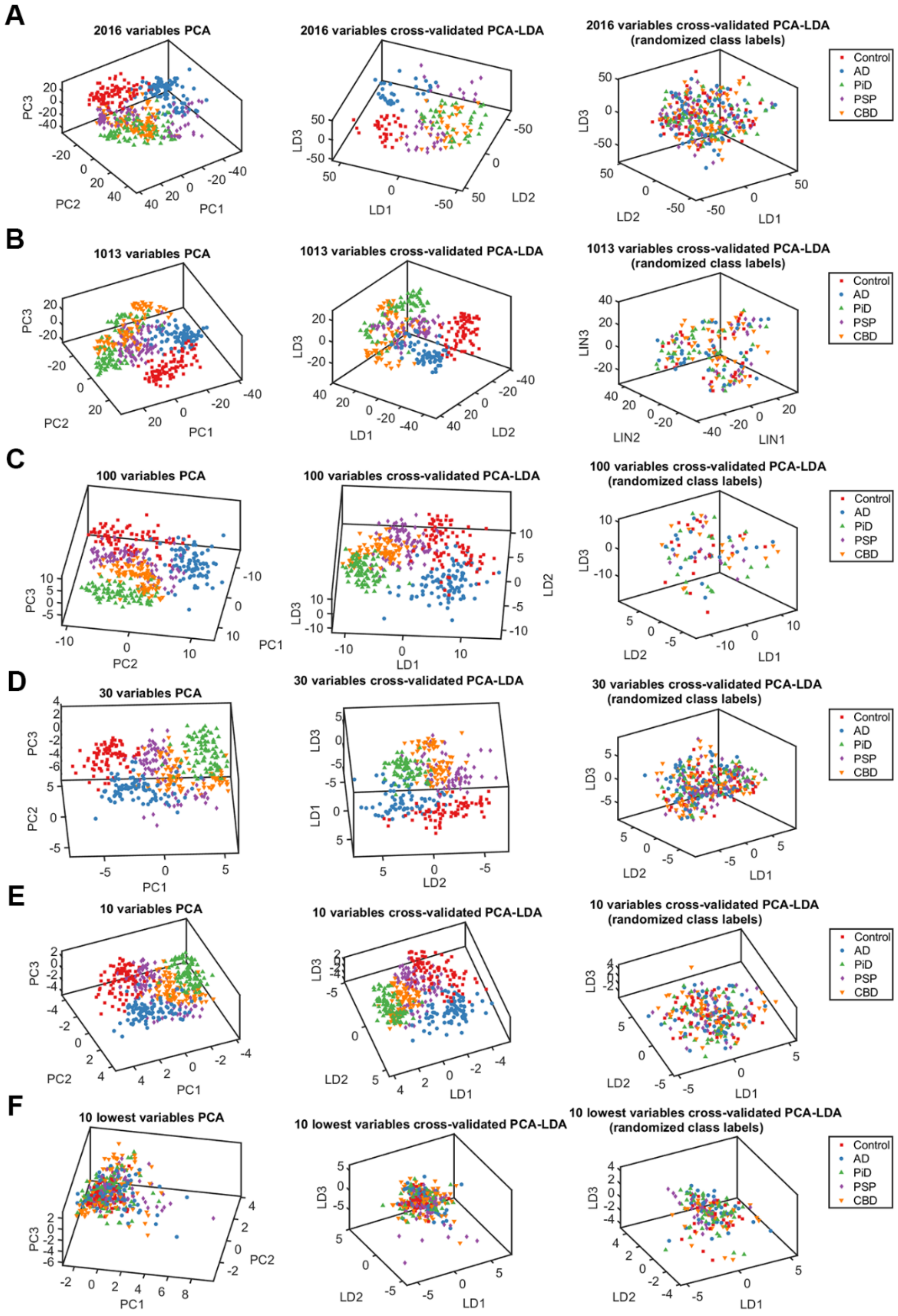

Figure S5. 100 features are optimal for clustering of MX-Raman barcodes

PCA analysis (left), PCA-LDA analysis (middle) and PCA-LDA analysis using randomized class labels (right) for MX-Raman spectra utilising A. All 2016 features of the MX-Raman fingerprint B. The 1013 highest ranking features of the MX-Raman fingerprint C. The 100 highest ranking features of the MX-Raman fingerprint D. The 30 highest ranking features of the MX-Raman fingerprint E. The 10 highest ranking features of the MX-Raman fingerprint F. The 10 lowest ranking features of the MX-Raman fingerprint. 3 PCs were retained for LDA and 5 k-folds were used for cross-validation. Class labels were also randomised before PCA-LDA to control for model overfitting (far right scatter plots). This data was used for Bhattacharyya distance analysis shown in Figure 3D of the main manuscript.

Detailed experimental methods

Raman acquisition

Each 785nm spectrum was collected for 45 s, with 15 s exposure to reduce fluorescence intensity to prevent detector saturation, followed by 2x 15 s measurement acquisitions. Each 532nm spectrum was collected for 30 s, with 10 s exposure to reduce fluorescence intensity to prevent detector saturation, followed by 4x 5 s measurement acquisitions. All raw spectra consisted of 1015 variables and detected using a Peltier cooled CCD (1024 pixels × 256 pixels) after dispersion through a diffraction grating (1 variable at each end of the spectrum was removed during preprocessing resulting in 1013 variables). 532nm spectra were diffracted through a 1200 L/mm grating with a range of 267 cm^{-1}-2005 cm^{-1} with a resolution of 1.71 cm^{-1} and 785nm spectra were diffracted through a 1800 L/mm grating with a range of 659 cm^{-1} –1761 cm^{-1} with a resolution of 1.09 cm^{-1}.

For Polarized Raman spectroscopy, a half wave plate was utilized to select a perpendicular/orthogonal polarization of the linearly polarized excitation laser before sample measurement and an analyzer was used to select parallel or orthogonal polarization of the Raman scattering from the sample. We measured spectra in parallel and perpendicular polarized detection conditions at the same locations on each sample. The depolarization spectra were obtained by taking the ratio of the raw perpendicular polarized spectra to the raw parallel polarized spectra (after quartz background subtraction).

For depolarization spectra, perpendicular polarized spectra were divided by parallel polarized spectra (after quartz background subtraction). Spectra were then processed in the same way as for normal Raman spectra. For autofluorescence spectra, Raman signals were removed by subtracting the processed Raman spectrum from its raw counterpart (after quartz background subtraction). Spectra were then processed in the same way as for normal Raman spectra, but without polynomial fluorescence removal.

Cluster analysis

To determine the spread of data in each cluster we used the Mahalanobis distance:

$$d = \sqrt{(y - \mu) \sum^{-1} (y - \mu)'}$$

Where d is the Mahalonobis distance from vector (y) to a distribution with a mean (µ) and covariance (∑). MX-Raman spectra transformed using PC1 and PC2 and each spectrum was then annotated with the corresponding class label. For each class, the Mahalanobis distance was calculated between each point and the centroid for that class and averaged. To determine cluster overlap, we used the Bhattacharyya distance:

$$d = \sqrt{(y - \mu) \sum^{-1} (y - \mu)'}$$

Where BC is the Bhattacharyya Coefficient, a measure of the overlap between two statistical samples or populations:

$$d = \sqrt{(y - \mu) \sum^{-1} (y - \mu)'}$$

Where P(x) and Q(x) are the discrete probability distributions along the x axis (i.e. along some single dimension). MX-Raman spectra transformed using PC1, PC2 and PC3 and each spectrum was then annotated with the corresponding class label. The Bhattacharyya distance was calculated between the ellipse of each class, referred to here as a cluster. 10 Bhattacharyya distances were measured from the pairwise distance measurement of the 5 clusters and averaged.

SDS-PAGE and Western Blot

The remaining sample was prepared for SDS-PAGE analysis. First, samples were sonicated for 25 s at 5% amplitude in cup horn sonicator (700W, 20kHz, Fisherbrand™) in polystyrene tubes at RT with 5x 5s cycles and 5s rest between each cycle. Sonicated samples were centrifuged for 30 s at 3000 x g. Samples were diluted 2 times into 2x concentrate Laemmli sample buffer (4% SDS, 20% glycerol, 0.004% bromphenol blue, 125 mM Tris-Cl, pH 6.8, 10% 2-mercaptoethanol) and boiled at 95 °C for 5 min. Samples were stored at -20°C until SDS-PAGE analysis.

Samples were thawed by boiling at 95 °C for 5min and separated by electrophoresis on mini-PROTEAN TGX Precast Protein Gels (4-20%, Tris-glycine, BioRad) at 200 V for 30 min. For western blotting, gels were transferred to PVDF membranes (Tran-Blot® TurboTM Mini PVDF Transfer packs, BioRad) using the Tran-Blot Turbo Transfer System (BioRad) and the preprogrammed 'mixed molecular weight protocol'. Membranes were incubated in blocking buffer (PBS, 0.1% TWEEN® 20, 5% BSA) for 1 h at RT and probed with primary antibody (Dako, tau, 1:15,000, rabbit polyclonal) overnight at 4°C with agitation followed by fluorescently-labelled secondary antibody (IRDye, LI-COR, 1:20,000, goat polyclonal) for 1 h at RT with agitation. Protein bands were measured using a LI-COR infrared scanner using the 800nm channel.

References

1. Devitt G, Rice W, Crisford A, Nandhakumar I, Mudher A, Mahajan S. Conformational Evolution of Molecular Signatures during Amyloidogenic Protein Aggregation. ACS Chemical Neuroscience. 2019;10(11):4593-611.
2. Devitt G, Crisford A, Rice W, Weismiller HA, Fan ZY, Commins C, et al. Conformational fingerprinting of tau variants and strains by Raman spectroscopy. Rsc Advances. 2021;11(15):8899-915.
3. Rygula A, Majzner K, Marzec KM, Kaczor A, Pilarczyk M, Baranska M. Raman spectroscopy of proteins: a review. Journal of Raman Spectroscopy. 2013;44(8):1061-76.
4. Spiro TG, Strekas TC. Resonance Raman-Spectra Of Heme Proteins - Effects Of Oxidation And Spin State. J Am Chem Soc. 1974;96(2):338-45.
5. Spiro TG. Resonance Raman-Spectroscopy as a Probe of Heme Protein-Structure and Dynamics. Adv Protein Chem. 1985;37:111-59.
6. Sato H, Chiba H, Tashiro H, Ozaki Y. Excitation wavelength-dependent changes in Raman spectra of whole blood and hemoglobin: comparison of the spectra with 514.5-, 720-, and 1064-nm excitation. J Biomed Opt. 2001;6(3):366-70.
